# Diversity of *Thelazia* spp. in domestic cattle from Romania: epidemiology and molecular diagnosis by a novel multiplex PCR

**DOI:** 10.1186/s13071-023-06012-8

**Published:** 2023-11-03

**Authors:** Vlad-Dan Cotuțiu, Angela Monica Ionică, Teodora Dan, Cristina Daniela Cazan, Silvia Diana Borșan, Carla Andreea Culda, Marian Mihaiu, Călin Mircea Gherman, Andrei Daniel Mihalca

**Affiliations:** 1 Department of Parasitology and Parasitic Diseases, University of Agricultural Sciences and Veterinary Medicine of Cluj-Napoca, Calea Mănăștur 3-5, 400372 Cluj-Napoca-Napoca, Romania; 2 Clinical Hospital for Infectious Diseases Cluj-Napoca, Iuliu Moldovan Street nr 23, Cluj-Napoca-Napoca, Romania

**Keywords:** *Thelazia* spp., *Bos taurus*, Multiplex PCR, Morphometry, Ecoregions

## Abstract

**Background:**

Thelaziosis is a neglected vector-borne disease caused by parasitic nematode worms of the genus *Thelazia* which affects various hosts. Limited attention has been given to ungulate-associated *Thelazia* species. Current diagnosis of thelaziosis and the identification/differentiation of species heavily relies on morphological features. Therefore, we conducted an epidemiological study in Romanian cattle, with the aim to obtain morphological and molecular data that can be used for species identification.

**Methods:**

The eyes of 705 slaughtered cattle were sampled and subjected to morphological identification, morphometric analysis, and molecular characterization. PCR amplification and sequence analysis were performed based on the cytochrome^c^ oxidase subunit 1 (COI) gene. Statistical tests assessed the correlations between infection parameters and ecological or biogeographical factors. A novel PCR method was developed based on the consensus sequence from each species. Specific forward primers were designed for each of the three species, and a reverse primer (COIintR) was used for all reactions. A consensus thermal profile was established by gradient PCR amplification of each species separately.

**Results:**

Of the sampled cattle, 19.3% were infected with *Thelazia* spp. Prevalence varied significantly with ecogeographical factors. A total of 585 *Thelazia* nematodes were recovered, with *T. rhodesi* being the most abundant, followed by *T. skrjabini* and *T. gulosa*. Morphometric and molecular analyses supported the morphological identification, yielding unique sequences for each species. From the 59 *T. rhodesi* specimens sequenced, 29 unique sequences were obtained, with a 99.1–99.85% nucleotide identity to the only other COI sequence present in GenBank®. All nine *T. gulosa* isolates were unique (99.37–100% nucleotide identity to other sequences), while *T. skrjabini* specimens displayed 98.47–100% nucleotide identity to the sole available sequence.

**Conclusions:**

Bovine thelaziosis is prevalent in Romania, raising concerns for animal welfare and potential economic impacts. Infected cattle grazing alongside vulnerable wild ruminants, such as the European bison, may affect conservation efforts. Our newly developed multiplex PCR shows promise as a valuable surveillance tool, enabling the detection of occult infections in apparently healthy animals through lachrymal secretion testing.

**Graphical Abstract:**

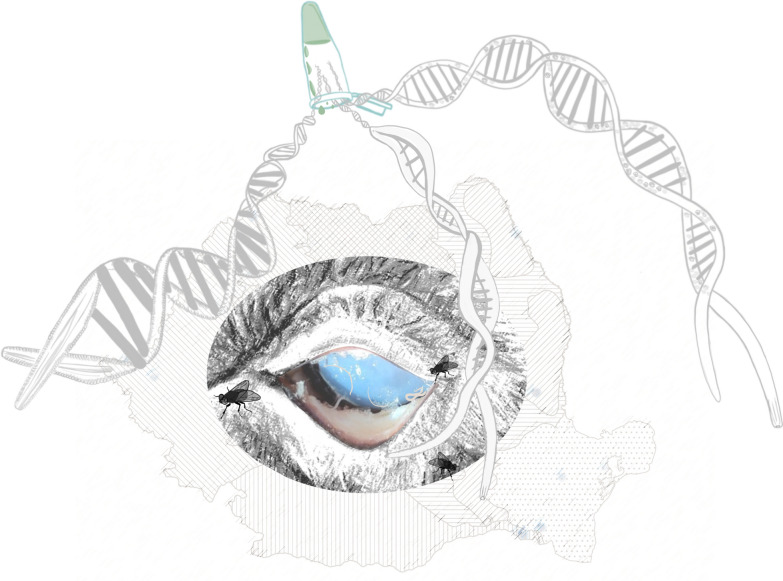

**Supplementary Information:**

The online version contains supplementary material available at 10.1186/s13071-023-06012-8.

## Background

Thelaziosis is a neglected vector-borne disease caused by parasitic nematode worms of the genus *Thelazia*. Adult nematodes of this genus infest the conjunctival sack of their host, while the first three larval stages develop in secretophagous flies. Parasites of this genus infect a wide range of wild and domestic mammals, including humans, and more rarely wild birds. Over the past century, the disease, especially in livestock and horses, has been thoroughly studied due to its relatively wide geographical distribution, which seems to be ubiquitous outside the polar regions, and impact on animal welfare [1–11]. The introduction of macrocyclic lactones in the 1980s seems to be associated with an apparent decrease in the number of reported cases of thelaziosis in livestock [12]. As a result, with the exception of a plethora of studies on the emergence of *Thelazia callipaeda* in Europe [13–20], over the past decades only a few studies have focused on *Thelazia* species associated with domestic and wild ungulates [7, 21–26]. Moreover, except for the well-documented zoonotic role of the carnivore-associated species *T. callipaeda* [27–29] and *Thelazia californiensis* [30], few studies have reported human infections caused by the cattle-associated *Thelazia gulosa* in the USA [31, 32].

Three *Thelazia* species (i.e. *T. rhodesi*, *T. skrjabini* and *T. gulosa*) are found in domestic and wild bovines in Europe. These are mainly vectored by the face fly *Musca autumnalis* [6], but other species of genus *Musca* are occasionally involved in the transmission of these nematodes [1]. However, the current epidemiology of bovine thelaziosis in Europe is poorly documented, with few published reports in the last decades. Similarly, in Romania, after the first reports of *Thelazia* species from the early and mid-twentieth century [33–35], there have been no studies until a recent clinical report [25].

The differentiation of these three cattle-associated species has been routinely based on morphological criteria. However, little information is currently available on the morphology of immature stages and sub-adults. Moreover, identification based on genetic analysis has been attempted only on a few occasions and then only on isolated cases [22, 25, 36, 37].

In this context, our aim was to perform an extensive cross-sectional epidemiological study in cattle slaughtered in Romania and to document new morphological and molecular data to facilitate the specific identification of species.

## Methods

### Sampling

Between January 2021 and June 2022, the eyes of 705 domestic bovines (*Bos taurus*, *n* = 698; *Bubalus bubalis*, *n* = 7) that had been slaughtered at six different abattoirs from Romania were collected. The following data were collected and recorded for each animal: species, sampling date, age, sex, geographic origin and breed (if available) (Additional file 1: Raw data of abattoir samples Spreadsheet). The sampling protocol used was as previously described [26].

### Morphology and morphometry

**Fig. 1:**
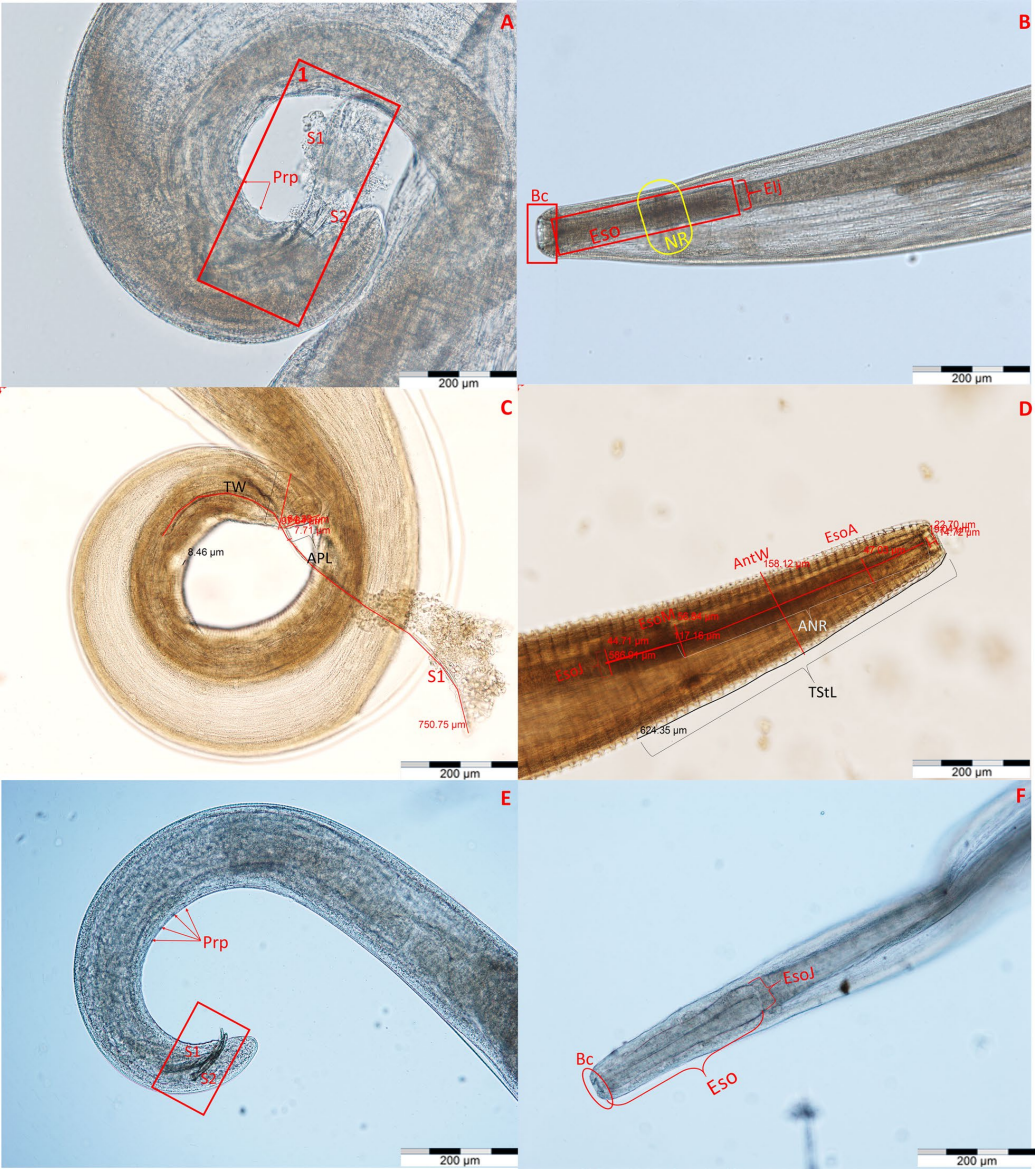
Morphological features of *Thelazia gulosa* (**a**, **b**), *Thelazia rhodesi* (**c**, **d**) and *Thelazia skrjabini* (**e**, **f**) males. **a** Tail of *T. gulosa* with two spicules (S1, S2), as well as the preanal papillae (Prp). **b** Proximal part of *T. gulosa* male, with the buccal capsule (Bc), esophagus (Eso) and nerve ring (NR) highlighted. **c** Tail of *T. rhodesi* with measurements of the longer spicule (S1), tail width near the anal pore (TW) and length from the anal pore to the tip of the tail. **d** Measurements of the parameters located in the proximal end of a *T. rhodesi* male, anterior width (AntW, halfway between the esophageal-intestinal junction and proximal buccal capsule), width of the esophagus at the junction with the intestine (EsoJ), maximum width of the esophagus (EsoM), anterior width of the esophagus (EsoA, the location of the measurement being the halfway point between the anterior width and the proximal width of the buccal capsule), total striation length (TStL, the distance is divided by the number of striations present) and the anterior nerve ring distance (ANR, the distance between the halfway point of the nerve ring and the proximal end of the esophagus). **e** Tail end of a *T. skrjabini* male presenting two spicules (S1, S2) and preanal papillae (Prp). **f** Proximal end of a *T. skrjabini* male with the buccal capsule (Bc), esophagus (Eso) and esophageal-intestinal junction (EsoJ) highlighted

**Fig. 2:**
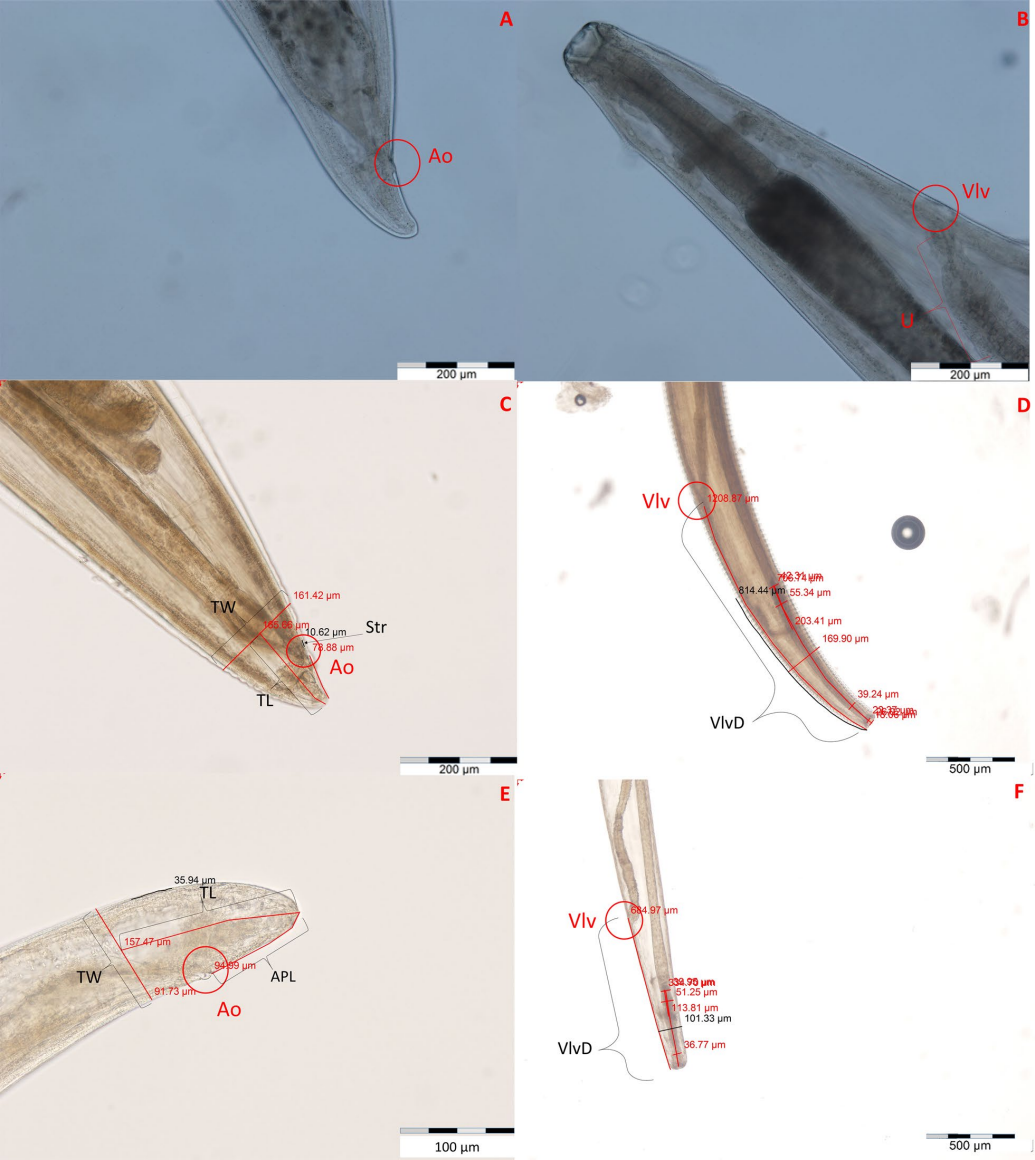
Morphological features of *Thelazia gulosa* (**a**, **b**), *Thelazia rhodesi* (**c**, **d**) and *Thelazia skrjabini* (**e**, **f**) females. **a** Tail of a *T. gulosa* female, with the anal pore encircled (Ao). **b** Proximal end of a *T. gulosa* female, with the genital pore (Vlv) and uterus (U) highlighted. **c** Tail of a *T. rhodesi* female, with measurements of tail width (TW), tail length (TL), striation size (Str). **d** Proximal end of a *T. rhodesi* female, with measurements including anterior width (AntW, halfway between the esophageal-intestinal junction and proximal buccal capsule), width of the esophagus at the junction with the intestine (EsoJ), maximum width of the esophagus (EsoM), anterior width of the esophagus (EsoA, the location of the measurement being the halfway point between the anterior width and the proximal width of the buccal capsule), total striation length (TStL, the distance is divided by the number of striations present) and the anterior nerve ring distance (ANR, the distance between the halfway point of the nerve ring and the proximal end of the esophagus) and the distance between the genital pore (Vlv) and the proximal width of the buccal capsule (VlvD). **e** Tail of a *T. skrjabini* with measurements of TW, TL and anal pore length (APL). **f** Proximal end of a *T. skrjabini* female with measurements of VlvD

**Fig. 3:**
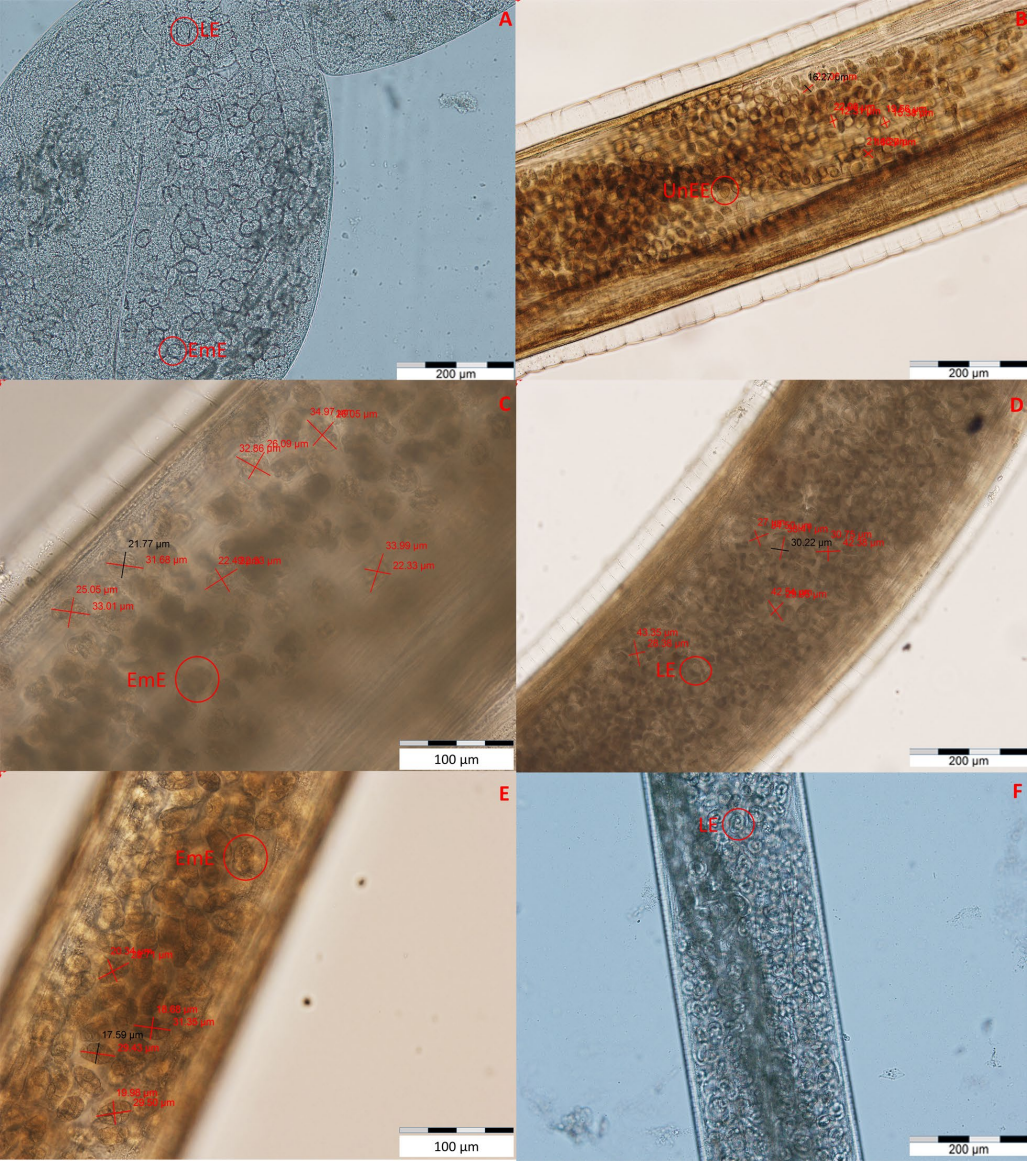
Morphology of larvae of *Thelazia gulosa* (**a**, **b**), *Thelazia rhodesi* (**c**) and *Thelazia skrjabini* (**d**).** a** Larvae (L) outside of the ruptured uterus of a *T. gulosa* female. **b** Larvae within the same *T. gulosa* female. **c** Larvae (L) within the uterus of a gravid *T. rhodesi* female. **d** Larvae (L) and eggs with larva (LE) within and outside of a *T. skrjabini* gravid female

**Fig. 4:**
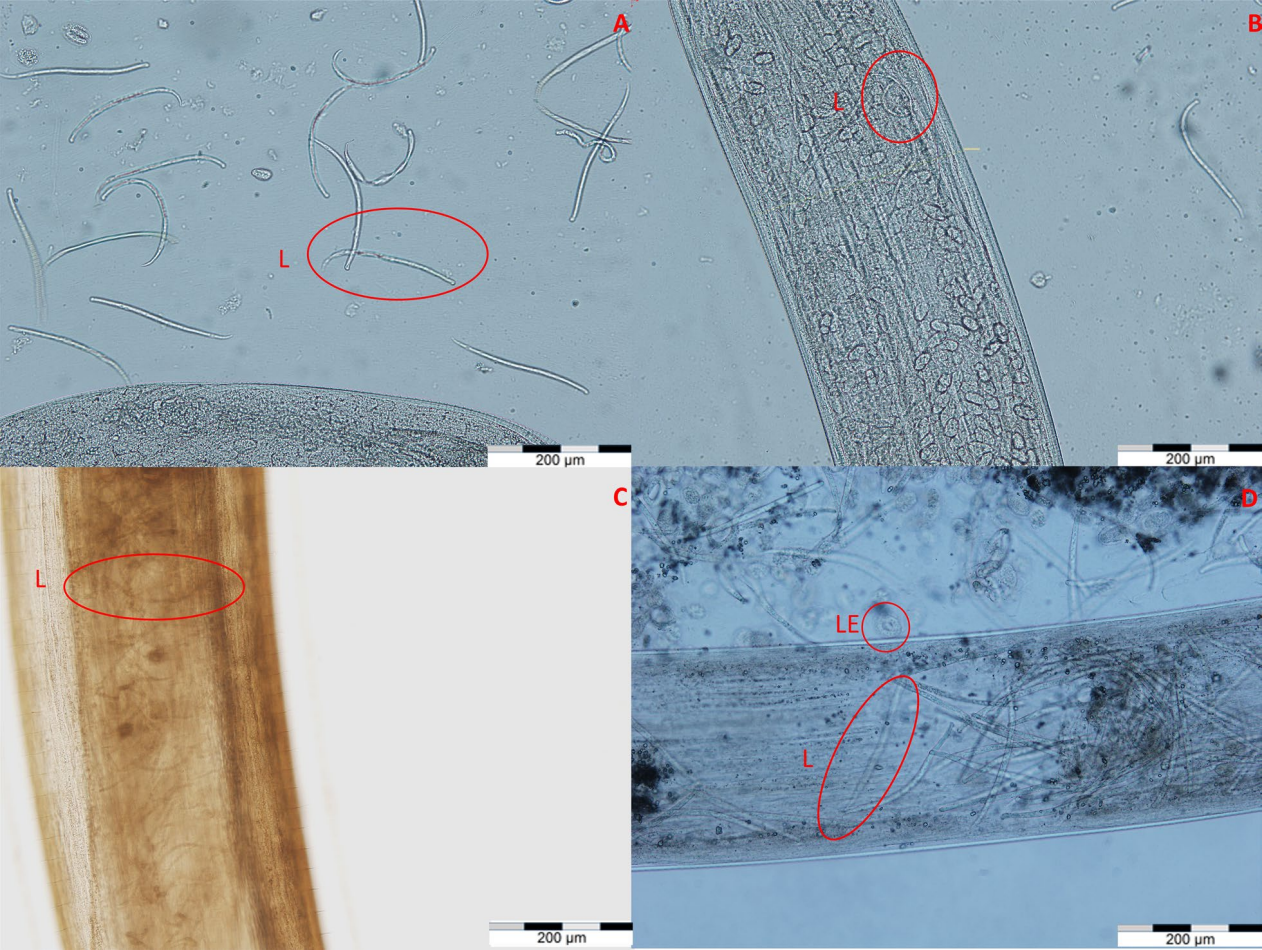
Morphology of eggs of *Thelazia gulosa* (**a**), *Thelazia rhodesi* (**b**–**d**) and *Thelazia skrjabini* (**e**) gravid females.** a** Gravid *T. gulosa* female with blastomeryzed eggs (EmE) and eggs containing first-stage larvae (LE). **b** Nonblastomeryzed eggs (UnEE) in a gravid *T. rhodesi* female **c**, **d** EmE and LE in *T. rhodesi*. **e** EmE and LE in *T. skrjabini*

Each individual nematode specimen was identified based on morphological characteristics according to developmental stage and the available keys [1, 6, 38]. A detailed morphometric analysis was performed on all undamaged nematodes, which included 18 parameters for fourth-stage larae (L4), 19 parameters for fifth-stage larvae (L5), 21 parameters for adult males and 27 parameters for adult females (Figs. 1, 2, 3, 4). The minimum/maximum and median values were calculated for each parameter. All measurements were made under an Olympus BX61 microscope (Olympus Corp., Tokyo, Japan supported by its dedicated software (Cell F version 3.1).

### Molecular characterization

#### DNA extraction, PCR amplification and sequence analysis

Genomic DNA was isolated from 87 specimens individually (59 *T. rhodesi*, 19 *T. skrjabini* and nine *T. gulosa*) using a commercial kit (ISOLATE II Genomic DNA Kit; Meridian Bioscience, London, UK). The nematodes were further analyzed by PCR amplification targeting an approximately 670-bp region of the mitochondrial cytochrome* c* oxidase I gene (*COI*) using the COIintF/COIintR primer pair, as described in [39]. All amplicons were purified (ISOLATE II PCR and Gel Kit; Meridian Bioscience) and sent to Macrogen Europe (Amsterdam, The Netherlands) for bidirectional sequencing. The chromatograms were assembled and edited using Geneious software (Biomatters Ltd., Auckland, New Zealand), and the consensus sequences obtained were compared to those of other *Thelazia* spp. isolates available in the GenBank® database by Basic Local Alignment Search Tool (BLAST) analysis. Calculation of genetic distances and the phylogenetic analysis were conducted using MEGA X software [40]. The analysis involved a total of 64 nucleotide sequences: 52 obtained during the present study, 11 sequences of *Thelazia* spp. retrieved from GenBank®, and one sequence of *Dirofilaria immitis* as the outgroup. The sequences were aligned using the MUSCLE algorithm, and the evolutionary history was inferred by using the maximum likelihood method and Tamura-Nei model [41]. A discrete Gamma distribution was used to model evolutionary rate differences among sites [5 categories (+ *G*, parameter = 0.2101)].

#### Multiplex PCR design and testing

Based on the consensus sequence obtained for each of the three species, specific forward primers were designed (Additional file 2: TIF file) following the criteria of Sharrocks [42]: T_rh_F (5′-CTTACTTTTAYGGCTTTGTTAATA-3′), T_Sk_F (5′-CCAGCCCGAAATGAGAATG-3′) and T_Gu_F (5′-GTTGGATCAAATGAGTATA-3′). The reverse primer COIintR (5′-ATAAGTACGAGTATCAATATC-3′) was used for all reactions. The consensus thermal profile was established by gradient PCR amplification of each species separately. Specificity and sensitivity tests were conducted in a final reaction volume of 25 μl containing 12.5 μl My Taq® Red PCR Mastermix (Meridian Bioscience), 1 μl (10 pmol) of each forward primer, 1 μl (30 pmol) reverse primer COIintR, 6.5 μl of PCR water and 2 μl of template DNA. The amplification profile was as follows: 5 min of initial denaturation at 95 °C; 35 cycles of denaturation at 95 °C for 45 s, annealing at 55 °C for 45 s and extension at 72 °C for 45 s; and a final extension at 72 °C for 5 min.

### Statistical analysis

The collected data were grouped into several categories. The average age, expressed in months, of infected and uninfected cattle was used for the analyses. Seasons were defined as spring (March–May), summer (June–August), autumn (September–November) and winter (December–February) for both 2021 and 2022 according to the sampling date. Data on geographical origin were separated into categories of biogeoregions, ecoregions and altitude intervals. The following biogeoregions present in Romania were included: Pannonian (P), Steppic (S), Alpine (A) and Continental ©). The ecoregions included in the study were: Pannonian Mixed (PMF), East European forest steppe (EEFS), Carpathian montane coniferous forests (CMCF), Central European mixed forests (CEMF), Pontic steppe (PS) and Balkan mixed forests (BMF). Altitude intervals were arbitrarily chosen in order to include comparable sample sizes as follow: 0–200, 201–400, 401–600, > 600 m a.s.l.

Statistical analysis was performed using the Chi-square (*χ*^2^) test for the following datasets: distribution of *Thelazia* spp. and *T. rhodesi* by season, average age, sex, biological ecoregions, ecoregions, altitude intervals and breed. Due to a low sample size, no statistical assessments were performed separately for *T. skrjabini* and *T. gulosa*. Chi-square was also used to assess the differences between the presence of *Thelazia* spp. in the left and right eyes. The intensity of infection according to season, ecoregion and altitude interval was assessed by means of analysis of variance (ANOVA) testing.

The correlations between the intensity of infection and season, ecoregion, altitude and altitude interval were evaluated using the Spearman’s rho or Pearson's correlation coefficient, as appropriate. The correlation between the occurrence of *Thelazia* spp. and season, ecoregion and altitude interval was assessed by the point-biserial coefficient.

The calculation of prevalence and 95% confidence intervals (95% CI) and the Chi-square and ANOVA tests were performed using EpiInfo 7 software (U.S. Centers for Disease Control and Prevention [CDC], Atlanta, GA, USA), and correlations were assessed using the free on-line tool available at https://www.statskingdom.com/.

### Mapping

Maps were constructed using the QGis version 3.26 software (2022; https://www.qgis.org/en/site/) using the following open-access layers: BioRegions2016 (https://www.eea.europa.eu/en) and Digital map of European ecological regions (https://www.eea.europa.eu/en). Results were exported to Microsoft Excel (Microsoft Corp., Redmond, WA, USA), using the intersect option.

## Results

### Epidemiology

Of the 705 domestic bovines sampled, 136 (19.3%) were infected by nematodes belonging to genus *Thelazia*, with 95 positive animals (69.9%) harboring a unilateral infection and the remaining 41 (30.2%) having an infection in both eyes. The left eye was significantly more frequently infected (*χ*^2^ = 19.72; *df* = 1; *P* < 0.0001) than the right eye (77.9% vs. 51.5% of positive animals). The prevalence of *Thelazia* nematodes across the different categories is shown in Table 1. Statistically significant differences in prevalence values were observed for season (*χ*^2^ = 88.67; *df* = 3; *P* < 0.0001) and altitude intervals (*χ*^2^ = 10.21; *df* = 3; *P* = 0.016) (Table 1). Significant differences were also found between infected and uninfected animals according to the average age (*P* = 0.017) (Additional file 3: Table 2). Significant differences were also found between breeds linked to a *Thelazia* spp. infection (*χ*^2^ = 14.62; *df* = 2; *P* = 0.0007). The breeds subjected to analysis were Bălțată Românească, Holstein and Mixed breeds, based on sample sizes adequate for testing.

**Table 1:** Prevalence of *Thelazia* spp. across the different statistical categories

Variable	Category	Sampled (*n*)	Positive for *Thelazia* spp. (*n*)	Prevalence of *Thelazia* spp. (%)	95% Confidence interval	*P*
Sex	Male	218	38	17.4	12.64–23.13	0.537
	Female	487	98	20.1	16.8–23.91	
Season	Spring	256	16	6.2	3.61–9.95	< 0.0001*
	Summer	310	62	20.0	15.93–24.81	
	Autumn	100	50	50.0	39.83–60.17	
	Winter	39	8	20.5	9.30–36.46	
Altitude intervals (m a.s.l.)	< 200	238	48	21.0	15.88–26.81	0.016*
	≥ 200 to < 400	229	49	20.6	15.64–26.29	
	≥ 400 to < 600	95	7	7.4	3.01–14.59	
	≥ 600	143	32	22.4	15.84–30.1	
Biogeoregion^a^	P	57	14	20.9	14.13–37.76	0.518
	S	101	20	19.8	12.54–28.91	
	A	164	35	20.3	15.34–28.41	
	C	383	67	16.9	14.02–21.62	
Ecoregion^b^	PMF	350	75	24.4	17.45–26.03	< 0.0001*
	BMF	7	6	85.7	42.13–99.64	
	PS	24	9	37.5	18.8–59.41	
	CMCF	176	35	19.9	14.26–26.56	
	EEFS	97	8	8.2	3.63–15.61	
	CEMF	51	3	5.9	1.23–16.24	
Total		705	136	19.3	16.55–22.37	

*Statistically significant

^a^Biogeregions: Pannonian (P), Steppic (S), Alpine (A) and Continental (C)

^b^Ecoregions: Pannonian Mixed (PMF), Balkan mixed forests (BMF), Pontic steppe (PS), Carpathian montane coniferous forests (CMCF), East European forest steppe (EEFS), Central European mixed forests (CEMF)

Of the correlations tested, the intensity according to season for both *Thelazia* spp. (*r* = 0.3; *P* < 0.0001) and *T. rhodesi* (*r* = 0.34; *P* < 0.0001) indicated a slight positive relationship. Moreover, the intensity according to ecoregions for *Thelazia* spp. (*r* = 0.007; *P* = 0.04) and *T. rhodesi* (*r* = 0.1; *P* = 0.007) also indicated the presence of a slightly positive relationship (Additional file 3: Tables 9, 14, 16, 25, 30, 32). The point-biserial correlation also indicated a slight positive and significant relationship between prevalence of *Thelazia* spp. and seasons (*r* = 0.28; *P* < 0.0001).

**Fig. 5:**
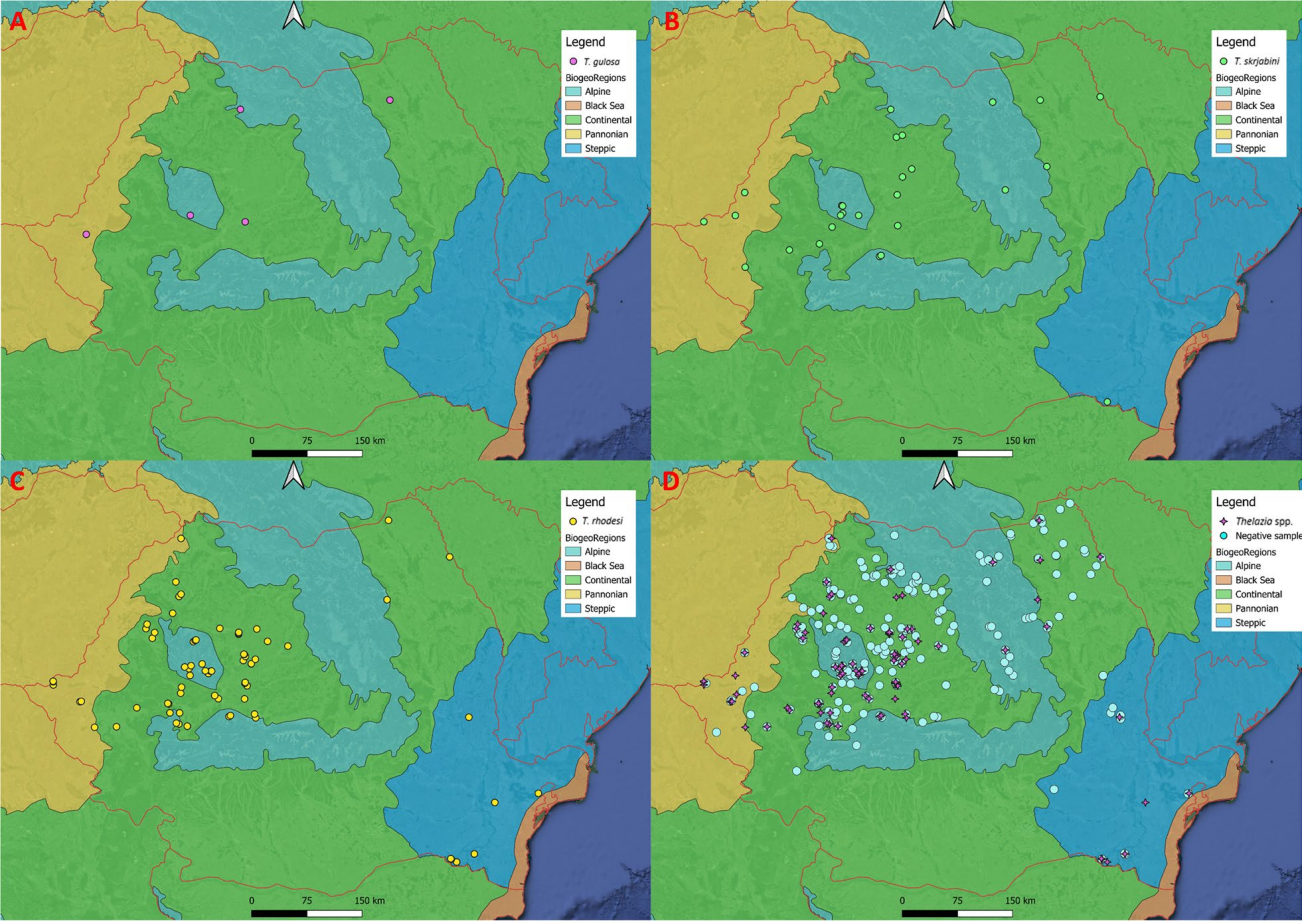
Distribution of *Thelazia* species according to biogeoregion. **a** *T. gulosa*, **b** *T. skrjabini*, **c** *T. rhodesi*, **d** *Thelazia* spp. and uninfected animals

**Fig. 6:**
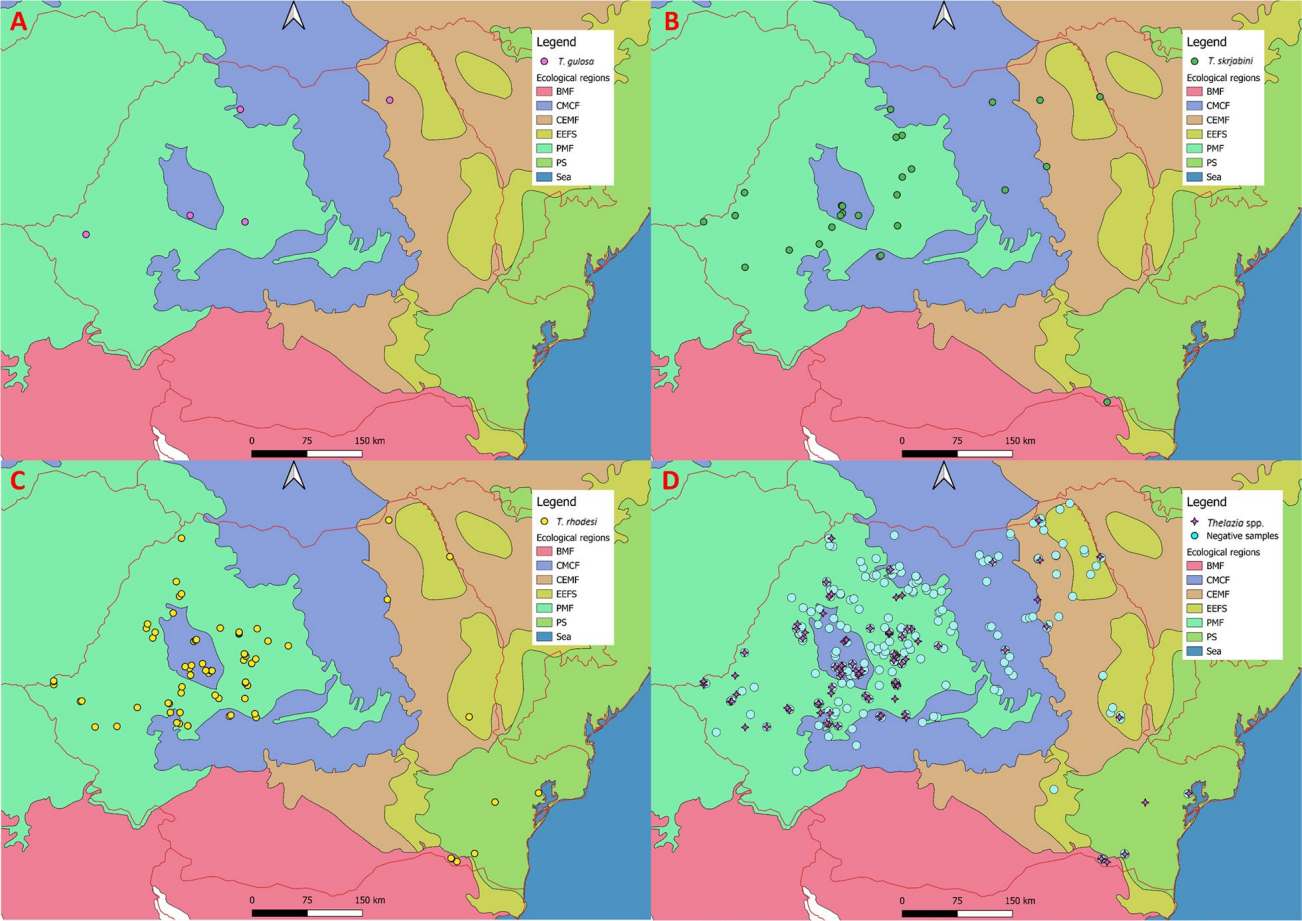
Distribution of *Thelazia* species according to ecoregion. **a** *T. gulosa*, **b** *T. skrjabini*, **c** *T. rhodesi*, **d** *Thelazia* spp. and uninfected animals

A total of 585 *Thelazia* spp. nematodes were recovered, with a mean intensity of infection of 4.3 (median = 3 nematodes/animal) (Table 2). *Thelazia rhodesi* was the most abundant of all *Thelazia* species, with 458 nematodes in different stages identified, followed by *T. skrjabini *(109 specimens) and *T. gulosa* (18 specimens). Also, a total of 11 co-infections were found, of which nine (81.81%) included *T. skrjabini* + *T. rhodesi*; the remaining two co-infections (18.18%) were with *T. skrjabini* + *T. gulosa*. There were no co-infections with *T. rhodesi* + *T. gulosa*. Maps on the distribution of *Thelazia* species are shown in Figs. 5 and 6.

**Table 2:** Intensity of *Thelazia* spp. infection per eye and by developmental stages of each species

*Thelazia* spp.	Intensity of infection^a^	Mean intensity of infection^b^	Minimum^b^	Maximum^b^	Median^b^
*T. rhodesi*					
Total* n*	458	4.5	1	41	3
M	102	0.9	0	15	0
F	288	2.5	0	26	2
L5	35	0.3	0	5	0
L4	33	0.3	0	4	0
*T. skrjabini*					
Total* n*	109	3.9	1	13	4
M	20	0.7	0	3	0
F	83	2.8	0	8	2
L5	4	0.1	0	1	0
L4	2	0.1	0	1	0
*T. gulosa*					
Total* n*	18	3.2	1	9	3
M	3	0.6	0	3	0
F	11	2.2	0	6	1
L5	4	0.8	0	2	0
L4	0	0	0	0	0
Total	585	4.3	1	41	3

*F* Adult females,* L4* fourth-stage larvae,* L5* fifth-stage larvae,* M *adult males

^a^Total number of *Thelazia* nematodes (in all different stages) recovered from all animals

^b^Mean, minimum, maximum and median number of nematodes per animal

### Morphometry

Morphometrical analysis is shown in Additional file 4: Raw data spreadsheet and Additional file 5: Min Max Med spreadsheet. Moreover, a value-based distinction was made between the egg types present in gravid females: The median measurements for larvated eggs measured were 39.2/29.4 µm for *T. rhodesi* and 41.8/28.0 µm for *T. skrjabini*. The medium measurements for blastomeryzed eggs were 32.8/23.6 µm for *T. rhodesi*, 30.8/21.7 µm for *T. skrjabini* and 18.3/13.9 µm for *T. gulosa*. Non-blastomeryzed eggs were only recorded in *T. rhodesi* (21.5/15.6 µm) and *T. skrjabini* (15.5/12.9 µm) (Fig. 4).

### Molecular analysis

The results of the genetic analysis were in complete agreement with the morphological identification of species. For *T. rhodesi*, from the 59 sequenced specimens analyzed, a total of 29 unique sequences were obtained, having a 99.1–99.85% identity to the only *T. rhodesi* COI sequence existing in GenBank® (Accession no. MT511659). All nine *T. gulosa* isolates were unique, having a 99.37–100% identity to the other two existing *T. gulosa* COI sequences (Accession nos. AJ544881, OL362019). From the 19* T. skrjabini* specimens, 14 unique sequences were obtained, having a 98.47–100% identity to the single *T. skrjabini* COI sequence from GenBank® (Accession no. OL362009). The identity of the sequences in our study was between 98.7 and 100% for *T. rhodesi*, 97.94–100% for *T. skrjabini* and 98.9–99.84% for *T. gulosa* (Additional file 6: Distance value comparison Spreadsheet).

**Fig. 7:**
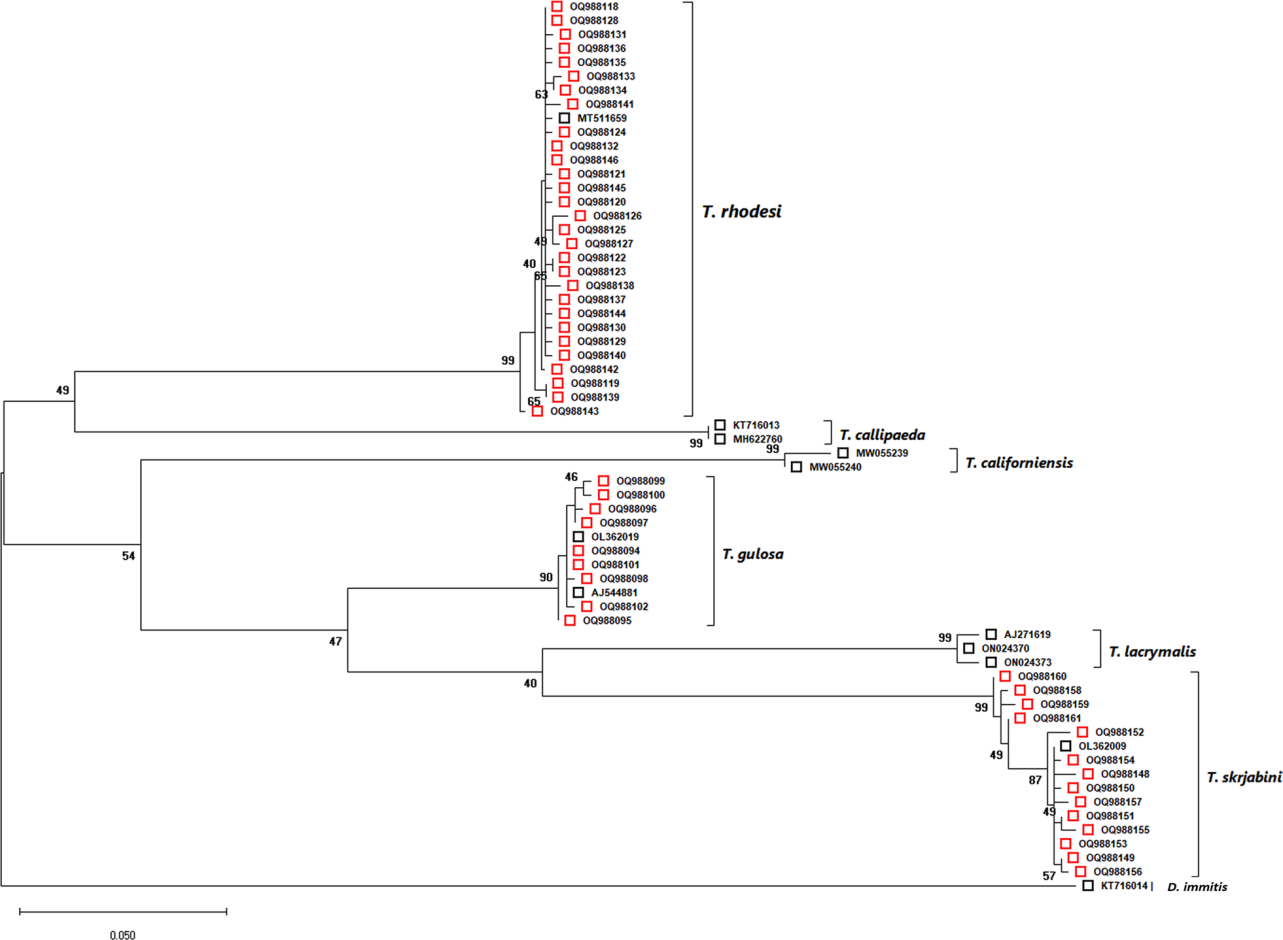
Bootstrap consensus tree inferred from 1000 replicates. The tree with the highest log likelihood is shown. The percentage of trees in which the associated taxa clustered together is shown next to the branches (values < 40% not shown). The tree is drawn to scale, with branch lengths measured in the number of substitutions per site. The red squares represent sequences obtained in the current study; the black squares represent existing sequences in GenBank

The sequences obtained during the current study were deposited in GenBank under the following accession numbers: OQ988094-OQ988102 for *T. gulosa*; OQ988118-OQ988146 for *T. rhodesi*; and OQ988148-OQ988161 for *T. skrjabini*. A bootstrap consensus tree was drawn, based on 62 nucleotide sequences, with a total of 528 positions in the final dataset (Fig. 7).

The multiplex PCR yielded bands of the expected sizes for all of the three species (approx. 360 bp for *T. gulosa*, approx. 480 bp for *T. skrjabini* and approx. 580 for *T. rhodesi*), both in single amplification and in various combinations (Additional file 7: Multiplex PCR validation ﻿TIF file).

## Discussion

The results of our study showed that bovine thelaziosis is a common, but seemingly neglected disease in Romania. In Romania, three *Thelazia* species (*T. skrjabini*, *T. gulosa* and *T. rhodesi*) have been identified, and these they can be differentiated by our novel multiplex PCR. Moreover, the current study represents the most detailed phylogenetic analysis of genus *Thelazia* performed up to date. The molecular analysis confirmed the identity of all three species based on morphological findings. From an evolutionary standpoint, *T. rhodesi* clusters together with *T. callipaeda*, while *T. gulosa* and *T. skrjabini* form a different clade, which also includes *T. lacrymalis* and *T. californiensis*. *Thelazia skrjabini* seems to be more closely related to the equine-specific species, *Thelazia lacrymalis* (Fig. 7; Additional file 6: Distance value comparison Spreadsheet).

Thelaziosis was a prominent disease of livestock in the Old World during the first half of the twentieth century, with the majority of reports originating in eastern European [43–45] and Mediterranean regions [46–48]. The second half of the century saw the disease’s distribution increase, ultimately spreading throughout most of the continent [1, 2, 48–53], with sporadic reports from Africa [54, 55], Asia [56], Australia [57] and the Americas [58]. In the past 40 years, coinciding with the wide use of macrocyclic lactones and the expansion of *T. callipaeda* in pets, wildlife and humans in Europe, thelaziosis in large herbivores has gone mostly unnoticed. The few reports that emerged [6, 12, 24–26, 59, 60] are a testament to its apparent decline. However, our data, obtained on an extensive dataset, are proof that thelaziosis is very much present, with almost one in the five cows tested in our study found to have harbored an infection with at least one species of *Thelazia*. In Romania, studies on the epidemiology of genus *Thelazia* in domestic cattle date back to the beginning of the 1970s [35], with a similar reported prevalence of 22.4%, as revealed also by eye examination in slaughterhouses in the north-eastern part of the country.

In our study, an effect of seasonality was evident, both for the prevalence and intensity of infection (Table 1). This is likely linked to the seasonality of the vector but also to the routine bi-annual deworming protocols performed by field veterinarians [61], which likely influenced the low prevalence levels in spring and early summer. Prevalence spikes occurred in late summer and autumn. The high prevalence in February could be attributed to the origin of the animals, most belonging to small individual extensive livestock production systems (Additional file 1: Raw data of abattoir samples Spreadsheet, Geographic origin tab). Such systems are subject to less restrictive regulations, leading to precarious hygiene, fewer preventive measures (ANSVSA president Order 208/2022 for implementing identification and registration of bovines, ovine, caprine, swine, camelids, cervinae and reindeer, https://legislatie.just.ro/Public/DetaliiDocumentAfis/263889) and higher parasitic prevalence and intensity [62–64].

Our statistical analysis revealed significant differences in the prevalence of *Thelazia* spp. between altitude intervals (Table 1; Additional file 3: Table 5). Consequently, altitude appears to act as a limiting factor, probably more so against the dipteran vectors rather than the parasite itself [65]. However, altitude seldom is the sole cause of a species inadaptability, whereas the associated environmental conditions (temperature, radiation output, precipitation and wind turbulence) appear to constitute a much more plausible explanation [66]. These factors could also explain the variability in the distribution of *Thelazia* spp. and its main vector, *M. autumnalis*, at different altitude levels, more specifically between 400 and 600 m a.s.l. The authors of several studies have concluded that wind speed is directly proportional with altitude [67, 68]. Nevertheless, a limitation of our study resides in the fact that > 50% of samples in the 400-to–600 m a.s.l. interval were collected in mid to late spring, when the prevalence was minimal (Additional file 1: Raw data of abattoir samples Spreadsheet, Date and Altitude Tab).

Specific ecological conditions (climate, habitat types or interactions with other organisms) (Fig. 6) may primarily shape the distribution of *Thelazia*. Both the overall prevalence of *Thelazia* spp. and that of *T. rhodesi* varied significantly according to ecoregion (Table 1). This variation could be related to different vector activity, seasonality or abundance [65] but also to the sample bias, as the BMF and PS ecoregions were sampled during the peak prevalence season (July–October), whereas the CEMF and EEFS ecoregions were mostly sampled in the spring and early summer. No statistically significant differences were found between biogeoregions (Fig. 5) for *Thelazia* spp. prevalence.

The overall intensity of infections varied greatly among animals, with a maximum value of 41 nematodes per animal (Table 2). The overall median intensity per animal was lower than that reported in a study from Italy [6] and in a previous study from Romania [35]. The median intensity of *T. skrjabini* was similar to values reported in Canada [69]. The highest intensity values were recorded in October and November and the lowest intensity was recorded in the spring, in line with previous records [35].

Co-infections with different *Thelazia* species seems to be rare (1.6%), as shown also by Dulceanu [35] and Moolenbeek and Surgeoner [58]. This is probably related to the evident dominance of *T. rhodesi* and the more sporadic occurrence of *T. skrjabini* and *T. gulosa* in Romania. These findings are similar to those reported in a study from Italy [6]; however, the prevalence and intensity of *T. skrjabini* in the current study was higher than that of *T. gulosa*. Demiaskiewicz et al. [24] also reported the presence of *T. gulosa* and *T. skrjabini* in European bison populations, with a higher intensity in *T. gulosa* than *T. skrjabini*. In North America, on the other hand, *T. gulosa* and *T. skrjabini* seem to be dominant, whereas *T. rhodesi* is absent [70].

Our morphometric analysis showed for the first time the size differences between the different egg types within the uterus of gravid *T. rhodesi*, *T. gulosa* and *T. skrjabini* females. This was previously shown in *T. callipaeda* [13]. Our study also provides, for the first time, the morphometric values for L4 and L5 of *T. skrjabini*, *T. rhodesi* and *T. gulosa*.

## Conclusions

Bovine thelaziosis seems to be relatively common in Romania, and most likely also in other geographical regions with similar ecological and animal husbandry conditions. Although clinical reports of thelaziosis are rare, the presence of ocular nematodes in cattle could represent an animal welfare concern, as well as having a negative economic impact due to decreased production. Moreover, the co-existence of infected cows with wild susceptible ruminants such as European bison (as the case of Romania which has three reintroduction sites) might impact the conservation efforts of the latter. The newly developed multiplex PCR described here could be a useful tool for the surveillance of occult infections in affected hosts by testing lachrymal secretions in apparently healthy animals.

### Supplementary Information

**Additional file 1: Excel spreadsheet (.xlsx).** Raw data of abattoir samples.

**Additional file 2:TAG Image file format (.tif).** Alignment of consensus sequences of the three *Thelazia* species and the position of the designed forward primers.

**Additional file 3: Excel spreadsheet (.xlsx).** Statistical tests performed on the database.

**Additional file 4: Excel spreadsheet (.xlsx).** Raw data of all measurements performed on recovered *Thelazia* specimens.

**Additional file 5: Excel spreadsheet (.xlsx). **Minimum, maximum and median values for all the parameters measured in the raw data file.

**Additional file 6: Excel spreadsheet (.xlsx).** Distance value comparison between original and available sequences.

**Additional file 7: TAG Image file format (.tif).** Multiplex PCR validation.

## Data Availability

All data generated or analysed during this study are included in this published article as well as its additional data. Sequences generated in this study are available in GenBank (OQ988094-OQ988102; OQ988118-OQ988146; OQ988148-OQ988161).

## Abbreviations

BMF: Balkan mixed forests
CEMF: Central European mixed forests
CMCF: Carpathian montane coniferous forests
EEFS: East European forest steppe
PMF: Pannonian mixed forests
PS: Pontic steppe
