# Supplementary figures and images for: Diversity of Thelazia spp. in domestic cattle from Romania: epidemiology and molecular diagnosis by a novel multiplex PCR
Source: Parasit Vectors. 2023 Nov 3;16:400. doi: 10.1186/s13071-023-06012-8 (PMC10623811; doi:10.1186/s13071-023-06012-8)

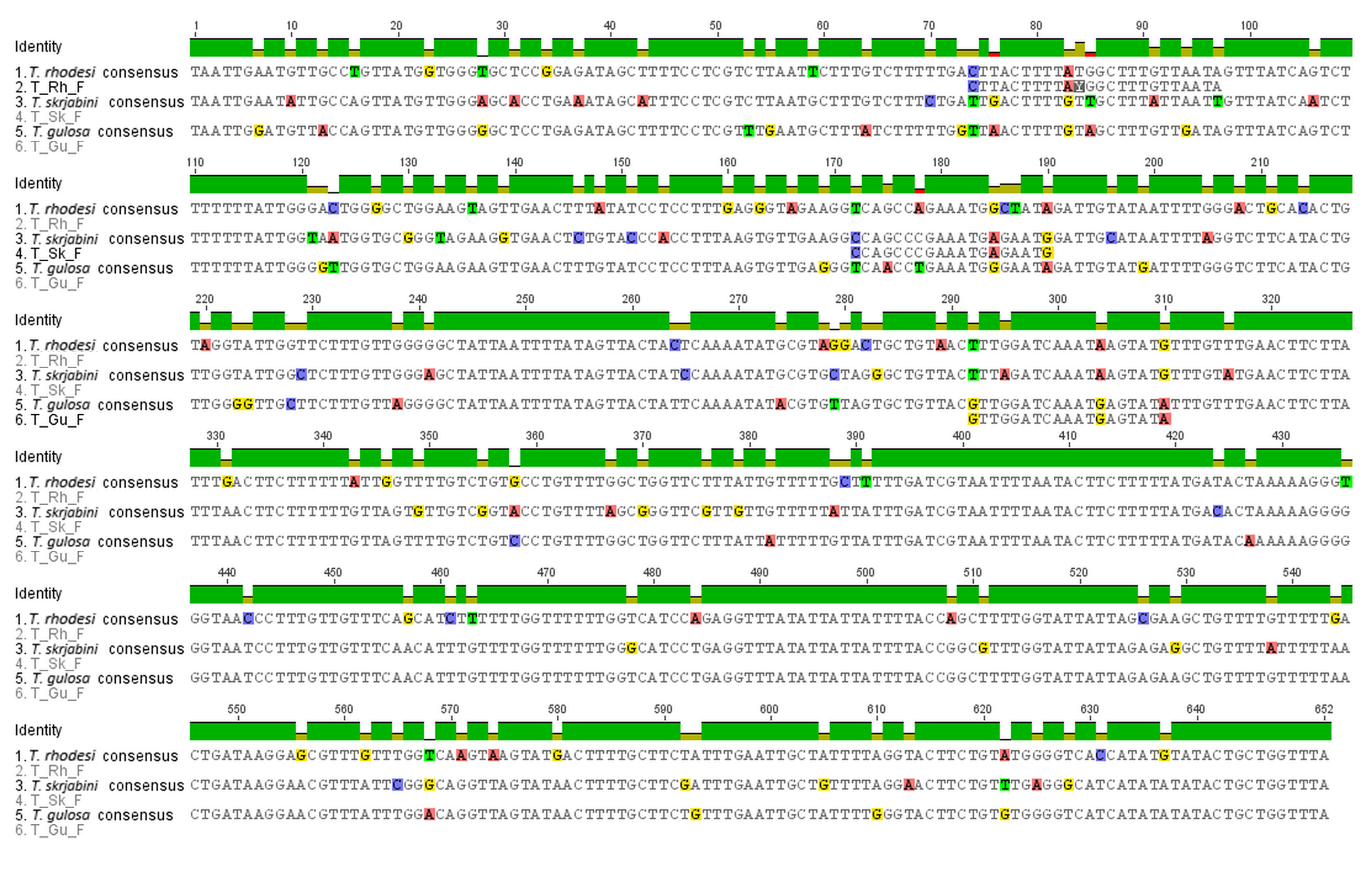

Supplement: Supplementary file 2 — Additional file 2:TAG Image file format (.tif). Alignment of consensus sequences of the three Thelazia species and the position of the designed forward primers. [file 13071_2023_6012_MOESM2_ESM.tif]

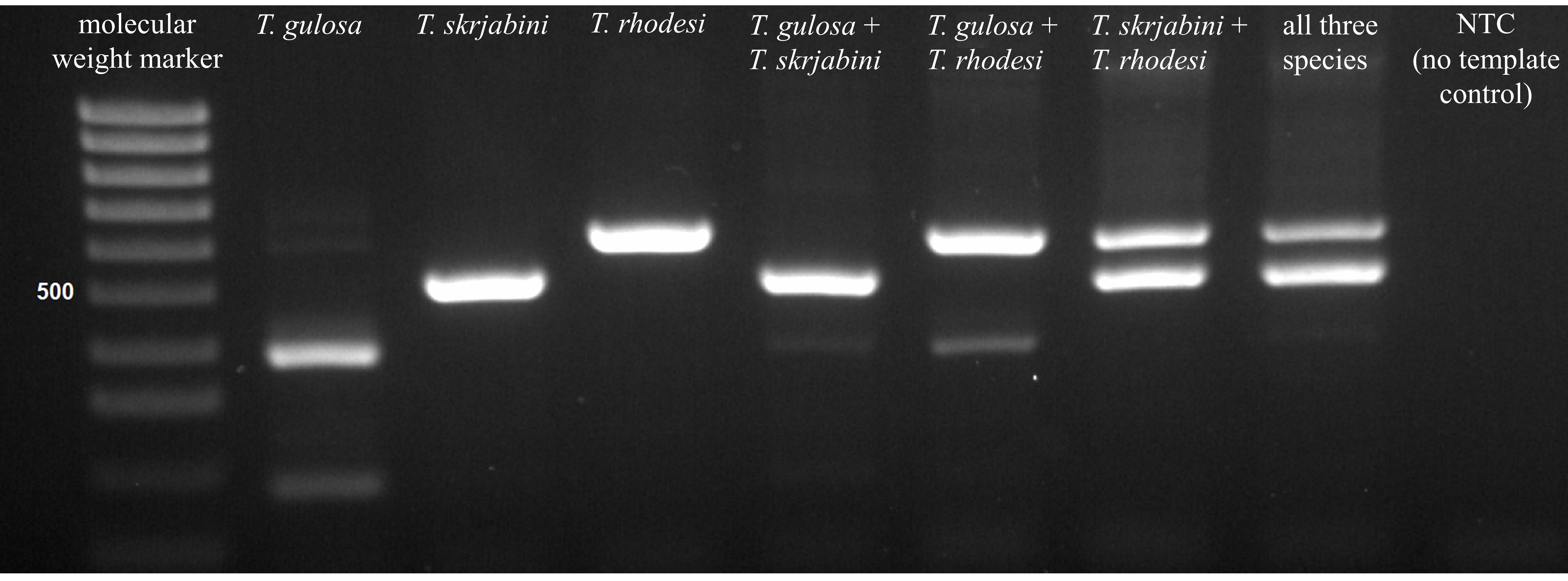

Supplement: Supplementary file 7 — Additional file 7: TAG Image file format (.tif). Multiplex PCR validation. [file 13071_2023_6012_MOESM7_ESM.tif]
